# The stealthy saboteur: hepatic steatosis and its erosion of APRI and FIB-4 accuracy

**DOI:** 10.3389/fmed.2026.1776500

**Published:** 2026-04-01

**Authors:** Huaying Zhou, Yuhong Li, Maoping Li, Jing Ma

**Affiliations:** Department of Infectious Diseases, The Second Xiangya Hospital, Central South University, Changsha, China

**Keywords:** APRI score, chronic hepatitis B, FIB 4, hepatic steatosis, liver biopsy, liver fibrosis

## Abstract

**Background and aims:**

Noninvasive models are increasingly important for liver fibrosis assessment. However, their accuracy can be affected by comorbidities. We aimed to evaluate the impact of concurrent hepatic steatosis (HS) on the diagnostic performance of the aspartate aminotransferase-to-platelet ratio index (APRI) and fibrosis-4 index (FIB-4) for staging liver fibrosis in patients with chronic hepatitis B (CHB).

**Methods:**

This retrospective cohort study included treatment-naïve CHB patients of Han Chinese ethnicity who underwent liver biopsy between January 2008 and December 2025. Patients were stratified into two groups: CHB without HS and CHB with HS. The diagnostic accuracy of APRI and FIB-4 for identifying advanced fibrosis (Metavir stage F3-F4) was assessed using the area under the receiver operating characteristic curve (AUROC) and at established clinical cut-offs.

**Results:**

In patients without HS, both APRI and FIB-4 demonstrated high diagnostic accuracy for advanced fibrosis, with AUROCs of 0.896 and 0.854, respectively. However, their performance was severely impaired by steatosis, with AUROCs dropping to just 0.473 for APRI and 0.468 for FIB-4 in patients with moderate-to-severe steatosis (S2-S3). This translated to a dramatic loss of clinical utility; for example, the positive predictive value (PPV) of APRI collapsed from 73.1% in the non-HS group to an unreliable 23.3% in the moderate-to-severe steatosis group.

**Conclusion:**

The presence of hepatic steatosis significantly compromises the diagnostic utility of APRI and FIB-4 for assessing advanced fibrosis in CHB patients. Clinicians should exercise caution when applying these noninvasive scores in CHB patients with known or suspected steatosis. We suggest prioritizing alternative methods, such as elastography-based techniques or newer biomarker panels, in this population. Our findings underscore the need for developing fibrosis models specifically validated or adjusted for patients with dual liver pathologies.

## Introduction

Hepatitis B virus (HBV) infection remains a major global health challenge, affecting an estimated 254 million people worldwide and leading to severe complications such as cirrhosis and hepatocellular carcinoma (HCC) (1, 2). Concurrently, the prevalence of metabolic dysfunction-associated steatotic liver disease (MASLD), formerly known as non-alcoholic fatty liver disease (NAFLD), is rising globally.

This convergence has led to a significant overlap, with studies estimating that concurrent hepatic steatosis (HS) affects 25% to over 50% of the global population with chronic hepatitis B (CHB), creating a growing population of patients with dual liver pathologies (3). The coexistence of CHB and hepatic steatosis (HS) is not a benign phenomenon; emerging evidence indicates that concurrent HS acts as an independent risk factor, potentiating HBV-associated HCC development and potentially impairing the response to antiviral therapies like entecavir (4). This clinical intersection underscores the urgent need for accurate disease staging in this specific patient subgroup.

Accurate assessment of liver fibrosis is paramount for guiding treatment decisions, predicting prognosis, and monitoring disease progression in CHB management (5, 6). While liver biopsy is considered the gold standard for staging fibrosis, its invasive nature, potential for severe complications, sampling variability, and inter-observer discrepancy limit its utility for routine clinical practice and longitudinal follow-up (7). Consequently, noninvasive fibrosis models have become indispensable tools in modern hepatology.

Among the most widely adopted models are the aspartate aminotransferase-to-platelet ratio index (APRI) and the fibrosis-4 index (FIB-4). Recommended by major clinical practice guidelines, these simple, cost-effective scores are calculated from routine laboratory parameters, making them highly accessible worldwide (5). While their diagnostic utility has been extensively validated in various chronic liver diseases, including CHB, these studies have predominantly been conducted in patient cohorts where comorbidities like HS were either not present or not accounted for as a significant variable (8).

This presents a critical knowledge gap. Hepatic steatosis itself is known to cause low-grade hepatic inflammation and can elevate aminotransferase levels, which are key components of both APRI and FIB-4 calculations (9). It is therefore plausible that the presence of HS could act as a significant confounder, distorting the scores and leading to an inaccurate estimation of fibrosis stage. To date, no large-scale study has systematically evaluated the impact of HS on the diagnostic performance of APRI and FIB-4 specifically within a biopsy-proven CHB population.

Therefore, the aim of this study was to compare the diagnostic accuracy of APRI and FIB-4 for identifying extensive liver fibrosis in a large cohort of CHB patients, stratifying the analysis based on the presence and severity of concurrent hepatic steatosis confirmed by liver biopsy.

## Materials and methods

### Study design and patient population

This retrospective cohort study was conducted at the Department of Infectious Diseases, the Second Xiangya Hospital of Central South University. All enrolled patients were of Chinese Han ethnicity. The study protocol was approved by the hospital’s Clinical Research Ethics Committee (Approval No. LYEC2025-K0004), which confirmed that the study involves human subjects and the requirement for individual patient consent was waived due to the retrospective nature of the analysis. We retrospectively identified treatment-naïve of Han Chinese ethnicity with chronic hepatitis B (CHB) who underwent a percutaneous liver biopsy between January 2008 and December 2025.

### Inclusion and exclusion criteria

Inclusion criteria were: (1) age ≥ 18 years; and (2) a confirmed diagnosis of CHB, defined as the presence of serum hepatitis B surface antigen (HBsAg) for at least 6 months, with HBV DNA > 2,000 IU/mL and elevated ALT/AST levels, consistent with the American Association for the Study of Liver Diseases (AASLD) guidelines for initiating treatment or performing a biopsy (10).

Exclusion criteria were: (1) co-infection with hepatitis C virus (HCV), hepatitis D virus (HDV), or human immunodeficiency virus (HIV); (2) other etiologies of chronic liver disease, such as autoimmune hepatitis, primary biliary cholangitis, primary sclerosing cholangitis, Wilson’s disease, or hemochromatosis; (3) a history of significant alcohol consumption, defined as a self-reported intake of > 20 g/day for females and > 30 g/day for males, as documented in patient interviews and physician’s notes. We acknowledge that this assessment was not verified by objective biomarkers such as phosphatidylethanol (PETH) (11, 12); (4) evidence of hepatocellular carcinoma (HCC) at the time of biopsy; (5) prior liver transplantation or antiviral therapy for HBV; and (6) decompensated cirrhosis.

### Data collection

Clinical and laboratory data were collected from electronic medical records within one week of the liver biopsy. Demographic data included age and sex, Body Mass Index (BMI), and history of type 2 diabetes. Laboratory parameters included complete blood count [platelet count (PLT), white blood cell count (WBC), red blood cell count (RBC), hemoglobin (Hb)], liver function tests [alanine aminotransferase (ALT), aspartate aminotransferase (AST), albumin (ALB), globulin (GLO), total bilirubin (TBil), direct bilirubin (DBil), total bile acid (TBA)], and serum HBV DNA levels, which were quantified by polymerase chain reaction (PCR).

### Histopathological assessment

All liver biopsy specimens were formalin-fixed, paraffin-embedded, and stained with hematoxylin-eosin and Masson’s trichrome. To be considered adequate for diagnosis, specimens were required to be at least 1.5 cm in length and contain a minimum of 6 portal tracts. For this study, all slides were re-evaluated by two experienced pathologists, who were blinded to the patients’ clinical and laboratory data, independently evaluated all slides. Any discrepancies in staging or grading were resolved by consensus discussion. The inter-observer agreement for fibrosis staging was excellent (Kappa statistic = 0.85).

Liver fibrosis was staged according to the Metavir scoring system: F0 (no fibrosis), F1 (portal fibrosis without septa), F2 (portal fibrosis with few septa), F3 (numerous septa without cirrhosis), and F4 (cirrhosis) (13). Advanced fibrosis was defined as a Metavir score of F3 or F4.

Hepatic steatosis (HS) was graded based on the percentage of hepatocytes containing fat droplets, according to the criteria established by Kleiner et al: Grade 0 (none, < 5%), Grade 1 (mild, 5–33%), Grade 2 (moderate, > 33–66%), and Grade 3 (severe, > 66%). Patients were stratified into a non-HS group (Grade 0) and an HS group (Grade 1–3) (14).

### Noninvasive fibrosis scores

APRI and FIB-4 scores were calculated using the following standard formulas: APRI = [(AST (IU/L)/ULN of AST)/PLT (10^9^/L)] × 100. The upper limit of normal (ULN) for AST was set at 40 IU/L; FIB-4 = [Age (years) × AST (IU/L)]/[PLT (10^9^/L) × √ALT (IU/L)] (15).

### Statistical analysis

Statistical analyses were performed using SPSS software (version 17.0) and SAS software (version 9.3). Continuous variables were expressed as mean ± standard deviation (M ± SD) or median (interquartile range, IQR) for non-normally distributed data, and compared using the independent samples *t*-test or Mann-Whitney U test, respectively. Categorical variables were presented as numbers or percentages and compared using the chi-squared (χ^2^) test.

The diagnostic performance of APRI and FIB-4 for predicting advanced fibrosis (F3-F4) was evaluated using receiver operating characteristic (ROC) curve analysis. The area under the ROC curve (AUROC) with its 95% confidence interval (CI) was calculated to assess the overall diagnostic accuracy. For each index, the optimal cut-off value was determined by maximizing the Youden index (Sensitivity +++ Specificity − 1). Sensitivity, specificity, positive predictive value (PPV), and negative predictive value (NPV) were calculated at these optimal cut-offs. In addition, diagnostic performance was also assessed using previously established clinical cut-offs for APRI (>1.0 to rule in, < 0.5 to rule out) and FIB-4 ( > 3.25 to rule in, < 1.45 to rule out). A two-sided *P*-value < 0.05 was considered statistically significant.

## Results

### Baseline characteristics of the study population

A total of 1,239 patients who underwent liver biopsy were included in the final analysis. The baseline demographic, clinical, and histological characteristics of the entire cohort are summarized in Tables 1, 2. Based on the data, the mean age of the population was approximately 26.3 years, with a predominance of male patients (1,029/1,239, 83.0%). The mean Body Mass Index (BMI) for the cohort was 24.5 ± 3.8 kg/m^2^, and the overall prevalence of diabetes was 15.0%. Histological assessment revealed that 550 patients (44.4%) had advanced liver fibrosis (fibrosis stage F3-F4), consistent with a high-risk tertiary care population. Hepatic steatosis (HS) was present in 248 patients (20.0%), of whom 173 (14.0% of total) had mild steatosis (S1) and 75 (6.0% of total) had moderate-to-severe steatosis (S2–S3).

**TABLE 1:** Clinical characteristics of 1,239 patients.

Variables	Non-HS	HS	*P-*values
Number	991	248	0.481
Gender (F/M)	165/826	45/203	
Age (yr)	26.32 ± 10.91	25.62 ± 10.51	0.523
Hb (g/L)	140.38 ± 42.72	142.81 ± 28.75	0.259
RBC (10^12^/L)	4.63 ± 0.80	4.79 ± 0.78	0.149
WBC (10^9^/L)	5.58 ± 1.66	5.87 ± 1.62	0.061
PLT (10^9^/L)	139.56 ± 73.43	217.17 ± 108.40	0.000
ALT (IU/L)	82.72 ± 62.43	121.28 ± 82.72	0.000
AST (IU/L)	26.32 ± 20.29	57.37 ± 42.39	0.000
ALB (g/L)	43.85 ± 18.13	46.14 ± 30.95	0.138
GLO (g/L)	29.83 ± 21.32	29.55 ± 30.44	0.960
TBil (μmol/L)	29.98 ± 46.09	22.86 ± 24.03	0.021
DBil (μmol/L)	15.71 ± 37.07	9.33 ± 12.48	0.009
TBA (μmol/L)	23.21 ± 42.72	24.49 ± 45.95	0.698
HBV-DNA (Lg)	3.06 ± 3.28	2.81 ± 3.25	0.080
BMI	23.9 ± 3.50	26.8 ± 4.10	<0.001
Diabetes(%)	13.6	20.6	0.012

Non-HS were patients diagnosed chronic hepatitis B virus without hepatic steatosis; HS were patients diagnosed chronic hepatitis B virus and hepatic steatosis. Hb, hemoglobin; RBC, red blood cell count; WBC, white blood cell count; PLT, platelet count; ALT, alanine aminotransferase; AST, aspartate aminotransferase; ALB, albumin; GLO, globulin; TBil, total bilirubin; DBil, direct bilirubin; TBA, total bile acid.

**TABLE 2:** Characteristics of 1,239 patients at different stage.

Variables	Non-HS			HS		
Fibrosis	0–2	3–4	*P* values	0–2	3–4	*P* values
Number	541	450		148	100	
Gender (F/M)	98/443	67/383	0.175	23/125	22/82	0.252
Age (yr)	25.37 ± 10.69	27.23 ± 10.04	0.157	24.86 ± 10.00	26.73 ± 11.17	0.180
Hb (g/L)	141.63 ± 4564	135.82 ± 25.20	0.011	144.82 ± 29.83	139.84 ± 36.94	0.181
RBC (10^12^/L)	4.82 ± 3.94	4.44 ± 3.81	0.004	4.85 ± 0.81	4.74 ± 0.79	0.289
WBC (10^9^/L)	6.89 ± 1.76	4.71 ± 2.94	0.017	6.13 ± 1.78	5.76 ± 1.61	0.087
PLT (10^9^/L)	160.00 ± 65.58	115.00 ± 51.23	0.000	225.00 ± 85.60	205.58 ± 78.85	0.095
ALT (IU/L)	75.00 ± 53.71	92.01 ± 65.13	0.010	118.50 ± 81.60	125.39 ± 92.70	0.437
AST (IU/L)	21.50 ± 16.76	32.12 ± 22.71	0.002	54.20 ± 43.39	62.06 ± 58.16	0.758
ALB (g/L)	44.40 ± 19.68	41.53 ± 7.19	0.000	44.53 ± 6.62	43.83 ± 6.18	0.392
GLO (g/L)	29.50 ± 23.18	30.46 ± 7.58	0.292	27.66 ± 5.64	27.86 ± 5.76	0.792
TBil (μmol/L)	26.67 ± 42.80	37.96 ± 41.16	0.001	23.02 ± 20.21	22.62 ± 28.88	0.904
DBil (μmol/L)	12.92 ± 28.36	25.29 ± 64.94	0.000	9.72 ± 11.77	8.76 ± 13.50	0.567
TBA (μmol/L)	22.01 ± 39.38	29.46 ± 56.79	0.030	11.66 ± 4.39	10.24 ± 3.91	0.015
HBV-DNA (Lg)	3.22 ± 3.29	1.99 ± 3.02	0.000	2.99 ± 3.34	2.52 ± 3.12	0.267

All the patients were divided two subgroups with significant and advanced fibrosis stage. One was fibrosis stage 0-2, and the other was fibrosis stage 3-4. And fibrosis stage 3-4 was defined advanced fibrosis. Non-HS were patients diagnosed chronic hepatitis B virus without hepatic steatosis; HS were patients diagnosed chronic hepatitis B virus and hepatic steatosis.

### Comparison of characteristics between with and without hepatic steatosis

To investigate the influence of steatosis, we stratified the cohort into a Non-HS group (*n* = 991) and HS group (*n* = 248). The baseline characteristics were compared between these two groups, as detailed in Tables 1, 2. Patients in the HS group exhibited significantly higher BMI (26.8 ± 4.1 vs. 23.9 ± 3.5 kg/m^2^, *P* < 0.001) and a higher prevalence of diabetes (20.6% vs. 13.6%, *P* = 0.012) compared to the Non-HS group.

As expected, laboratory markers of liver injury were significantly elevated in the HS group, including mean ALT (121.28 ± 82.72 vs. 82.72 ± 60.75 IU/L, *P* < 0.001) and AST (57.37 ± 42.39 vs. 26.32 ± 20.29 IU/L, *P* < 0.001). Notably, the mean platelet count was also significantly higher in the HS group (217.17 ± 88.40 vs. 139.56 ± 62.43 × 10^9^/L, *P* < 0.001), a trend that deviates from the typical decline of platelets seen in advancing fibrosis. Furthermore, patients in the HS group showed significantly lower levels of total bilirubin (TBil, 22.86 vs. 29.98 μmol/L, *P* = 0.021) and direct bilirubin (DBil, 9.33 vs. 15.71 μmol/L, *P* = 0.009).

Critically, there were no significant differences in age (25.62 vs. 26.32 years, *P* = 0.523), sex distribution (*P* = 0.481) between the Non-HS and HS groups. As detailed in Table 2, the prevalence of advanced fibrosis (F3-F4) was also comparable between the two groups (40.3% in HS group vs. 45.4% in Non-HS group, *P*-value not shown but derivable), providing a solid baseline for assessing the impact of steatosis itself on the performance of non-invasive fibrosis models, without the confounding effect of disparate fibrosis distributions.

All the patients were divided two subgroups with significant and advanced fibrosis stage. One was fibrosis stage 0–2, and the other was fibrosis stage 3–4. And fibrosis stage 3–4 was defined advanced fibrosis. Non-HS were patients diagnosed chronic hepatitis B virus without hepatic steatosis; HS were patients diagnosed chronic hepatitis B virus and hepatic steatosis.

### Impact of hepatic steatosis on the diagnostic performance of APRI and FIB-4

Our primary analysis evaluated the ability of APRI and FIB-4 to identify advanced fibrosis (F3–F4) in patients stratified by the presence or absence of HS. In the Non-HS cohort (*n* = 991), both models demonstrated strong diagnostic performance. The Area Under the Receiver Operating Characteristic curve (AUROC) for APRI was 0.896 (95% CI: 0.865–0.927), and for FIB-4, it was 0.854 (95% CI: 0.812–0.896) (Figure 2 and Table 3). At its optimal cut-off of 2.06, APRI achieved a high negative predictive value (NPV) of 90.0%, establishing it as a reliable tool for ruling out advanced fibrosis in this population. Similarly, FIB-4 showed an excellent NPV of 93.1% at its optimal cut-off of 3.4.

**TABLE 3:** Impact of hepatic steatosis on the diagnostic performance of APRI and FIB-4 for advanced fibrosis (F3–F4).

Index	Group	AUROC (95% CI)	Cut-off	Sen (%)	Spe (%)	PPV (%)	NPV (%)
APRI	Non-HS	0.896 (0.865–0.927)	2.06	82.6	86.8	55.4	90
	HS	0.516 (0.398–0.634)	1.88	31.7	74.9	20.6	30.8
FIB-4	Non-HS	0.854 (0.812–0.896)	3.4	76.5	81.3	48.2	93.1
	HS	0.525 (0.406–0.644)	3.25	53.7	61.4	22.1	86.9

AUROC means area under receiver operator characteristic curve; CI means Confidence Interval. Non-HS means CHB without fatty liver, HS means fatty liver. Sen, sensibility; Spe, specificity; PPV, positive predictive value; NPV, negative predictive value.

**FIGURE 1:**
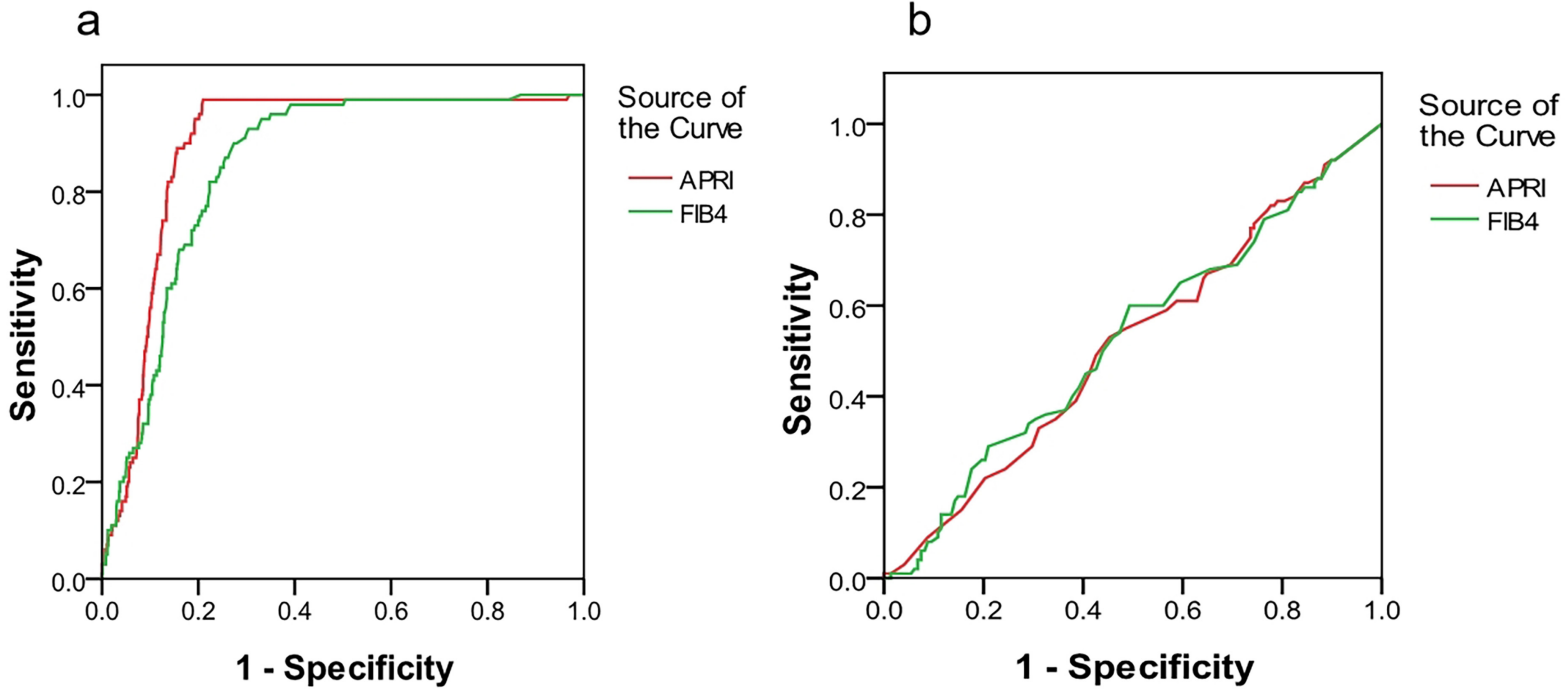
**(a,b)** The ROC of APRI and FIB 4 in CHB patients with/without HS with advanced fibrosis stage, respectively.

In stark contrast, the diagnostic utility of both models was substantially diminished in the presence of concurrent HS. The AUROCs plummeted to levels consistent with chance, measuring only 0.516 (95% CI: 0.398–0.634) for APRI and 0.525 (95% CI: 0.406–0.644) for FIB-4 (Figure 1 and Table 3). The clinical consequence of this performance degradation was profound: the NPV of APRI for excluding advanced fibrosis dropped precipitously from 90.0% in the Non-HS group to a mere 30.8% in the HS group, indicating a high risk of false-negative results. While FIB-4 maintained a higher NPV of 86.9% in the HS group, this was achieved at a very low specificity (61.4%) and positive predictive value (22.1%), limiting its clinical usefulness.

**FIGURE 2:**
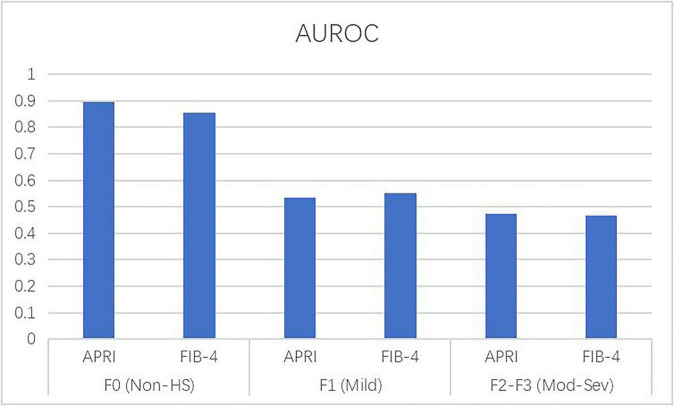
Distribution of fibrosis stages stratified by the presence and severity of hepatic steatosis. Data are presented as relative proportions (percentages) within each subgroup to facilitate comparison between cohorts. Fibrosis stages (F0–F4) are indicated by the color-coded segments. Total patient numbers for each segment are: Non-HS (*n* = 991), HS (*n* = 248), S1 (*n* = 173), S2 (*n* = 53), and S3 (*n* = 22). HS, hepatic steatosis; S1, mild steatosis; S2, moderate steatosis; S3, severe steatosis.

### Graded impairment of diagnostic accuracy by steatosis severity

**FIGURE 3:**
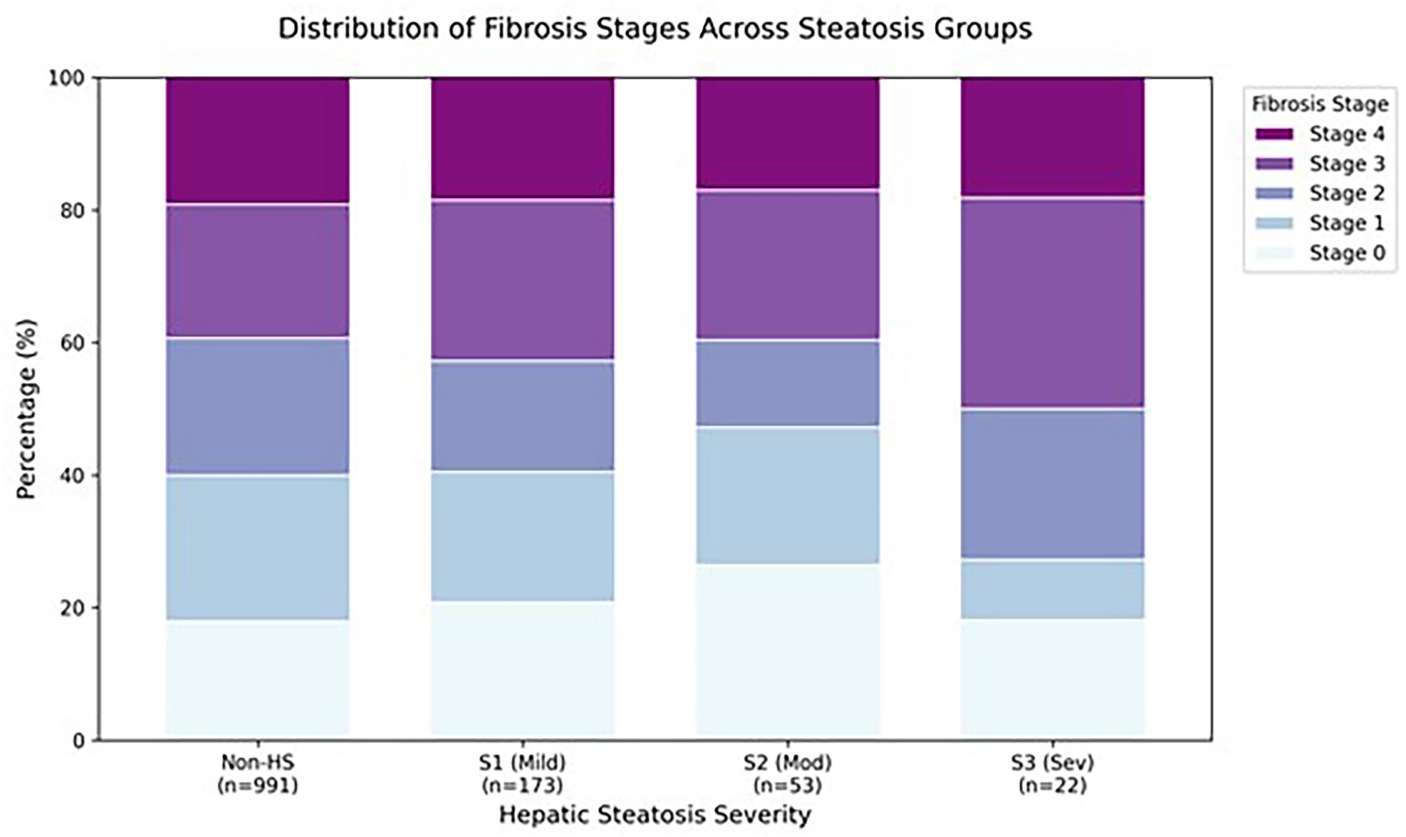
Decline in diagnostic accuracy with increasing severity of hepatic steatosis.

To further investigate this confounding effect, we performed a sub-analysis, stratifying the cohort by the histological grade of steatosis: Non-HS (S0, *n* = 991), mild steatosis (S1, *n* = 173), and moderate-to-severe steatosis (S2-S3, *n* = 75). The results revealed a clear severity-dependent relationship: the diagnostic accuracy of both APRI and FIB-4 progressively deteriorated as the severity of steatosis increased (Figures 2, 3 and Table 4).

**TABLE 4:** Diagnostic performance of APRI and FIB-4 for advanced fibrosis (F3-F4), stratified by steatosis severi.

Steatosis Grade	Index	AUROC (95% CI)	Cut-off	Sen (%)	Spe (%)	PPV (%)	NPV (%)
S0 (Non-HS)	APRI	0.896 (0.865–0.927)	1.05	87.2	75.6	73.1	88.7
*n* = 991	FIB-4	0.854 (0.812–0.896)	1.35	83.9	74.2	68.9	87.1
S1 (Mild HS)	APRI	0.536 (0.401–0.671)	0.95	58.3	55.4	33.7	77.8
*n* = 173	FIB-4	0.551 (0.415–0.687)	1.15	66.7	50.9	34.8	79.4
S2-S3 (Mod-Sev HS)	APRI	0.473 (0.288–0.658)	1.05	45.5	48.6	23.3	72
*n* = 75	FIB-4	0.468 (0.282–0.654)	1.25	45.5	54.1	26.3	74.1

AUROC means area under receiver operator characteristic curve; CI means Confidence Interval. CHB + mild HS was patients diagnosed chronic hepatitis B virus mild fatty liver; CHB + moderate HS was patients diagnosed chronic hepatitis B virus moderate fatty liver; CHB severe HS was patients diagnosed chronic hepatitis B virus moderate fatty liver. Sen, sensibility; Spe, specificity; PPV, positive predictive value; NPV, negative predictive value.

For APRI, the AUROC declined from 0.896 (95% CI: 0.865–0.927) in patients without steatosis (S0) to 0.536 (95% CI: 0.401–0.671) in those with mild steatosis (S1), and further to 0.473 (95% CI: 0.288–0.658) in those with moderate-to-severe steatosis (S2-S3). This decline in overall accuracy translated to a dramatic loss of clinical utility at their respective cut-offs. For instance, in the Non-HS group, APRI demonstrated a high Negative Predictive Value (NPV) of 88.7% and a solid Positive Predictive Value (PPV) of 73.1%. However, in the moderate-to-severe steatosis group, these values collapsed to an NPV of 72.0% and a clinically unreliable PPV of just 23.3%. This decline in overall accuracy translated to a dramatic loss of clinical utility. This indicates that in the presence of significant fat, more than three-quarters of patients identified as “high risk” by APRI would actually be false positives.

A parallel and equally pronounced decline was observed for FIB-4, with the AUROC dropping from 0.854 (95% CI: 0.812–0.896) in the S0 group to 0.551 (95% CI: 0.415–0.687) in the S1 group and ultimately to 0.468 (95% CI: 0.282–0.654) in the S2-S3 group. The clinical reliability of FIB-4 was similarly eroded. In the Non-HS group, FIB-4 showed a strong NPV of 87.1% and a PPV of 68.9%. In stark contrast, for patients with moderate-to-severe steatosis, the PPV fell to a mere 26.3%, rendering the model ineffective for confirming advanced fibrosis in this population. The progressive decrease in NPV from 87.1% (S0) to 74.1% (S2–S3) further underscores that FIB-4 loses its “rule-out” capability as steatosis worsens.

These findings confirm that hepatic steatosis acts not merely as a binary confounder but as a graded inhibitor of the diagnostic accuracy of these commonly used fibrosis scores. The presence of steatosis, particularly at moderate-to-severe levels, severely limits their ability to reliably rule in or rule out advanced fibrosis.

## Discussion

In this large-scale, biopsy-proven cohort of treatment-naïve CHB patients, our study provides robust evidence that the diagnostic accuracy of widely used non-invasive fibrosis models, APRI and FIB-4, is significantly compromised by the presence and severity of hepatic steatosis. We demonstrated that while both models performed excellently in patients without steatosis (AUROC for APRI: 0.896), their performance deteriorated dramatically in patients with even mild steatosis, and became worse than random chance (AUROC dropping to as low as 0.468) in those with moderate-to-severe steatosis. This severity-dependent impairment highlights a critical limitation of these scores in the increasingly prevalent population of patients with co-existing liver fibrosis and steatosis, a group that now frequently includes CHB patients with metabolic comorbidities (4, 8).

The underlying mechanism for this compromised performance likely stems from the core components of these indices. Both APRI and FIB-4 heavily rely on aminotransferase levels (AST and ALT), which are known to be disproportionately elevated or even paradoxically normal in patients with significant steatosis, irrespective of the underlying fibrosis stage (3, 16). Hepatic steatosis itself can induce a state of chronic, low-grade inflammation and cellular stress, leading to fluctuations in AST/ALT that do not correlate linearly with the progression of fibrosis (17). Consequently, the signal-to-noise ratio is disrupted; the “signal” from fibrosis-driven liver injury is drowned out by the “noise” from steatosis-related metabolic dysfunction. A particularly noteworthy finding from our baseline data was the significantly higher platelet count in the HS group, which contradicts the expected trend of lower platelets in more advanced liver disease. This anomaly further confounds FIB-4, which uses platelets in its denominator, potentially contributing to its failure in this subgroup. Our findings align with previous smaller studies but provide definitive, large-scale evidence of this phenomenon, underscoring that AST and ALT are unreliable surrogates for fibrosis in the context of significant steatosis (18).

The clinical implications of our study are profound. Relying on APRI and FIB-4 to screen for advanced fibrosis in populations with a high prevalence of steatosis, such as individuals with metabolic syndrome or type 2 diabetes, carries a substantial risk of misclassification. Our data highlight a dramatic inflation of false-positive results, leading to a collapse in positive predictive value and potentially triggering unnecessary, costly, and invasive follow-up procedures such as liver biopsies. This phenomenon is consistent with previous observations that co-existing fatty liver in CHB patients significantly confounds liver status assessment (19), and aligns with broader evidence indicating that non-invasive biomarkers often suffer from sub-optimal PPV in metabolic-related liver diseases, leading to diagnostic overestimation (20). Such diagnostic inaccuracies undermine clinical decision-making and erode patient trust. Furthermore, the poor specificity in these subgroups could lead to an inefficient allocation of medical resources, while any concurrent misclassification of true-positive cases risks delayed diagnosis, missed opportunities for intervention, and unmonitored progression to cirrhosis and its complications.

It is crucial to recognize that steatosis is not the only factor that can compromise the reliability of these widely used indices. The performance of APRI and FIB-4 is also influenced by acute inflammatory flares and, as recently highlighted by Ozdemir et al. (21), the patient’s antiviral treatment status. Their study found that while APRI and FIB-4 were moderately effective in treatment-naïve CHB patients, their utility was substantially diminished in post-treatment settings, a finding attributed to the decoupling of inflammation (ALT levels) from underlying fibrosis severity (21). This aligns with our findings in a different context: just as antiviral therapy alters the inflammatory component of the scores, hepatic steatosis introduces its own “inflammatory noise” by elevating AST levels, thereby misleading the indices and uncoupling them from the true extent of fibrosis.

Based on our findings, we propose a more cautious, stratified approach to clinical practice: when significant steatosis is known or suspected (e.g., via ultrasound or other biomarkers), clinicians should exercise extreme caution when interpreting APRI and FIB-4 scores (13). In this scenario, these scores should not be used as standalone diagnostic tools. Instead, the preferred clinical pathway should directly involve more robust, steatosis-independent non-invasive tests, such as vibration-controlled transient elastography (VCTE, which also provides a quantitative measure of steatosis via CAP) or magnetic resonance elastography (MRE), to accurately stage fibrosis (5).

A major strength of our study is its large sample size with liver biopsy as the gold standard, allowing for robust subgroup analyses based on the histological severity of steatosis (6). However, several limitations must be acknowledged. First, this was a retrospective, single-center study focused on a CHB cohort, which may limit the generalizability of our findings to other populations with different etiologies of liver disease (e.g., pure MASLD or alcohol-related liver disease). Second, while biopsy is the gold standard, it is subject to sampling error and inter-observer variability, a well-documented challenge in the ongoing debate between invasive and non-invasive assessment methods (22). Third, our study did not include a head-to-head comparison with elastography-based methods (e.g., VCTE, MRE) within the same cohort, which would have provided a direct quantification of the added value of these advanced techniques in patients with steatosis (22, 23). Future prospective, multi-center studies are needed to validate our findings and compare a wider array of non-invasive tests.

In conclusion, our study demonstrates that the utility of APRI and FIB-4 as frontline tools for fibrosis assessment is critically dependent on the patient’s steatosis status. In an era where metabolic dysfunction is increasingly intertwined with various chronic liver diseases, our work, together with findings like those from Ozdemir et al., underscores a broader principle: the accuracy of simple biomarker scores is context-specific, a new generation of fibrosis biomarkers that are robust against such metabolic and therapeutic confounders is urgently needed. Future prospective, multi-center research should focus on developing and validating such novel scores, potentially incorporating steatosis-specific markers or entirely new analytes, to improve the accuracy of non-invasive fibrosis staging in this large and growing patient population.

## Data Availability

The raw data supporting the conclusions of this article will be made available by the authors, without undue reservation.

## References

[1] Chan AW Wong GL Chan HY Tong JH Yu YH Choi PC et al. Concurrent fatty liver increases risk of hepatocellular carcinoma among patients with chronic hepatitis B. *J Gastroenterol Hepatol.* (2017) 32:667–76. 10.1111/jgh.13536 27547913

[2] World Health Organization [WHO] *Global Hepatitis Report 2024: Action for Access in Low-and Middle-income Countries.* Geneva: World Health Organization (2024).

[3] Huang SC Liu CJ. Chronic hepatitis B with concurrent metabolic dysfunction-associated fatty liver disease: challenges and perspectives. *Clin Mol Hepatol.* (2023) 29:320–31. 10.3350/cmh.2022.0422 36726053 PMC10121303

[4] Yu MW Lin CL Liu CJ Yang SH Tseng YL Wu CF. Influence of metabolic risk factors on risk of hepatocellular carcinoma and liver-related death in men with chronic hepatitis B: a large cohort study. *Gastroenterology.* (2017) 153: 1006–17.e5. 10.1053/j.gastro.2017.07.001. 28711626

[5] Terrault NA Lok ASF McMahon BJ Chang KM Hwang JP Jonas MM et al. Update on prevention, diagnosis, and treatment of chronic hepatitis B: aasld 2018 hepatitis B guidance. *Hepatology.* (2018) 67:1560–99. 10.1002/hep.29800 29405329 PMC5975958

[6] Younossi ZM Otgonsuren M Henry L Venkatesan C Mishra A Erario M et al. Association of nonalcoholic fatty liver disease (NAFLD) with hepatocellular carcinoma (HCC) in the United States from 2004 to 2009. *Hepatology.* (2015) 62:1723–30. 10.1002/hep.28123 26274335

[7] Rockey DC Caldwell SH Goodman ZD Nelson RC Smith AD American Association for the Study of Liver Diseases Liver biopsy. *Hepatology.* (2009) 49:1017–44. 10.1002/hep.22742 19243014

[8] Xiao G Yang J Yan L. Comparison of diagnostic accuracy of aspartate aminotransferase to platelet ratio index and fibrosis-4 index for detecting liver fibrosis in adult patients with chronic hepatitis B virus infection: a systemic review and meta-analysis. *Hepatology.* (2015) 61:292–302. 10.1002/hep.27382 25132233

[9] European Association for the Study of the Liver EASL 2017 clinical practice guidelines on the management of hepatitis B virus infection. *J Hepatol.* (2017) 67:370–98. 10.1016/j.jhep.2017.03.021 28427875

[10] Honar N Jooya P Haghighat M Imanieh MH Dehghani SM Zahmatkeshan M et al. Complications of blind versus ultrasound-guided percutaneous liver biopsy in children. *Arab J Gastroenterol.* (2015) 16:90–3. 10.1016/j.ajg.2015.09.009 26526508

[11] Viel G Boscolo-Berto R Cecchetto G Fais P Nalesso A Ferrara SD. Phosphatidylethanol in blood as a marker of chronic alcohol use: a systematic review and meta-analysis. *Int J Mol Sci.* (2012) 13:14788–812. 10.3390/ijms131114788 23203094 PMC3509610

[12] Perilli M Toselli F Franceschetto L Cinquetti A Ceretta A Cecchetto G et al. Phosphatidylethanol (PEth) in blood as a marker of unhealthy alcohol use: a systematic review with novel molecular insights. *Intern J Mol Sci.* (2023) 24:12175. 10.3390/ijms241512175 37569551 PMC10418704

[13] The French METAVIR Cooperative Study Group Intraobserver and interobserver variations in liver biopsy interpretation in patients with chronic hepatitis C. *Hepatology.* (1994) 20:15–20. 10.1002/hep.1840200104 8020885

[14] Kleiner DE Brunt EM Van Natta M Behling C Contos MJ Cummings OW et al. Design and validation of a histological scoring system for nonalcoholic fatty liver disease. *Hepatology.* (2005) 41:1313–21. 10.1002/hep.20701 15915461

[15] Ma J Jiang Y Gong G. Evaluation of seven noninvasive models in staging liver fibrosis in patients with chronic hepatitis B virus infection. *Eur J Gastroenterol Hepatol.* (2013) 25:428–34. 10.1097/MEG.0b013e32835cb5dd 23358121

[16] Lee BP Dodge JL Terrault NA. Reply: recognizing the new nomenclature requires a comprehensive analysis of the prevalence, severity, and long-term outlook for SLD and subclassifications. *Hepatology.* (2024) 79:E117–8. 10.1097/HEP.0000000000000661 37906684

[17] McPherson S Hardy T Henderson E Burt AD Day CP Anstee QM. Evidence of NAFLD progression from steatosis to fibrosing-steatohepatitis using paired biopsies: implications for prognosis and clinical management. *J Hepatol.* (2015) 62:1148–55. 10.1016/j.jhep.2014.11.034 25477264

[18] Yang A Nguyen M Ju I Brancatisano A Ryan B van der Poorten D. Utility of Fibroscan XL to assess the severity of non-alcoholic fatty liver disease in patients undergoing bariatric surgery. *Sci Rep.* (2021) 11:14006. 10.1038/s41598-021-93294-6 34234198 PMC8263818

[19] Fang X Yin Y Zhao H Wang C Li H Shang Y et al. Effect of fatty liver disease on liver function and fibrosis in patients with chronic hepatitis B: a cross-sectional study. *Front Med.* (2024) 11:1481051. 10.3389/fmed.2024.1481051 39640976 PMC11617145

[20] Vilar-Gomez E Chalasani N. Non-invasive assessment of non-alcoholic fatty liver disease: clinical prediction rules and blood-based biomarkers. *J Hepatol.* (2018) 68:305–15. 10.1016/j.jhep.2017.11.013 29154965

[21] Ozdemir YE Ozdemir MS Bayramlar OF Kaya SY Karaali R Balkan II et al. Performance of noninvasive fibrosis indices in chronic hepatitis B during pretreatment and post-treatment periods. *Biomark Med.* (2023) 17:799–809. 10.2217/bmm-2023-0405 38179991

[22] Borcak D Yesilbag Z Ozdemir YE Demir AS Dogdas ES Sezen AI et al. Assessing liver fibrosis in chronic hepatitis b: liver biopsy or non-invasive fibrosis markers? *J Clin Med.* (2025) 14:8164. 10.3390/jcm14228164 41303199 PMC12653223

[23] Harrison SA Ratziu V Boursier J Francque S Bedossa P Majd Z et al. A blood-based biomarker panel (NIS4) for non-invasive diagnosis of non-alcoholic steatohepatitis and liver fibrosis: a prospective derivation and global validation study. *Lancet Gastroenterol Hepatol.* (2020) 5:970–85. 10.1016/S2468-1253(20)30252-1 32763196

